# Modeling the infection risk and emergency evacuation from bioaerosol leakage around an urban vaccine factory

**DOI:** 10.1038/s41612-023-00342-1

**Published:** 2023-02-17

**Authors:** Zhijian Liu, Hongwei Cao, Chenxing Hu, Minnan Wu, Siqi Zhang, Junzhou He, Chuan Jiang

**Affiliations:** 1 School of Energy and Power Engineering, North China Electric Power University, Baoding, 071003 China; 2 School of Mechanical Engineering, Beijing Institute of Technology, Beijing, 100081 China

**Keywords:** Atmospheric dynamics, Environmental impact

## Abstract

Mounting interest in modeling outdoor diffusion and transmission of bioaerosols due to the prevalence of COVID-19 in the urban environment has led to better knowledge of the issues concerning exposure risk and evacuation planning. In this study, the dispersion and deposition dynamics of bioaerosols around a vaccine factory were numerically investigated under various thermal conditions and leakage rates. To assess infection risk at the pedestrian level, the improved Wells-Riley equation was used. To predict the evacuation path, Dijkstra’s algorithm, a derived greedy algorithm based on the improved Wells-Riley equation, was applied. The results show that, driven by buoyancy force, the deposition of bioaerosols can reach 80 m on the windward sidewall of high-rise buildings. Compared with stable thermal stratification, the infection risk of unstable thermal stratification in the upstream portion of the study area can increase by 5.53% and 9.92% under a low and high leakage rate, respectively. A greater leakage rate leads to higher infection risk but a similar distribution of high-risk regions. The present work provides a promising approach for infection risk assessment and evacuation planning for the emergency response to urban bioaerosol leakage.

## Introduction

Urban areas are where wealth and economies are concentrated, but they are also densely populated^1^. In the context of the COVID-19 pandemic, infectious research on virus-carrying bioaerosol particles (BPs) is no longer limited to indoor transmission, and mounting studies now focus on outdoor infections among urban residents by considering various transmission pathways of contagious BPs. For instance, residents were reported to have contracted COVID-19 from the air while farming outdoors (http://henan.china.com.cn/m/2022-04/13/content_41937765.html, in Chinese). In 2019, BPs that were not completely inactivated due to expired disinfectant water were accidently released from a vaccine factory in Lanzhou, China, resulting in the spread of active Brucella virus aerosols into nearby urban areas. Consequently, a biosafety incident occurred, leaving many people infected with the Brucella virus including the students in the research institute 400 m away as well as residents in the surrounding areas (http://wjw.lanzhou.gov.cn/art/2020/9/15/art_4531_928158.html, in Chinese). In addition, the United States and many other countries have also incurred terrorist and *Bacillus anthracis* attacks in their urban cities^2^. Evidently, urban areas with their crowded populations and complex geographic topology are at high risk of virus transmission and unexpected infection. In particular, a formidable challenge lies in predicting and responding to the short-term, large-scale transmission of infectious BPs released from vaccine factories, wastewater plants, or livestock farms.

Generally, computation fluid dynamics (CFD) simulation has been widely applied in research on urban building ventilation and pollutant dispersion, with wind tunnel experiments used to validate the simulation results^3,4^. Compared with Gaussian and integral models, an advantage to using CFD is that it can account for various meteorological conditions, chemical reactions, and complex urban environments^5,6^. It is proven that the aerodynamic behavior of airborne bioaerosols particles (organic or not) around building complexes can be well represented via either the Reynolds-averaged-Navier-Stokes (RANS) simulation approach or Large Eddy Simulations (LES)^7–9^. In particular, the flow fields operating at a urban scale can be reliably expressed by RANS simulations with a low consumption of computation power and reasonable accuracy in resolving turbulence^7,10–12^. For the modeling of a pollutant, both Euler and Lagrangian discrete phase models have been widely applied, but they often yield different results in terms of its dynamic concentration^13^. An idealized urban model was built and numerically simulated using the Euler model to investigate urban ventilation and pollutant dispersion under different building densities and aspect ratios^14^. In other work, a tracer gas was used to replicate the process of a virus leaking from a ventilation shaft and spreading into an adjacent tall building^15^. The simulation results obtained via the Euler model are capable of incorporating well the real exposure risks between the adjacent tall buildings. Yet, because there exists an interaction force between particles and air, particles could also affect the flow field to a certain extent. Meanwhile, the deposition of particles on the surfaces of certain buildings should not be neglected, especially when an outdoor-to-indoor transmission is possible. Accordingly, the Lagrangian particle-tracking model, which can fully consider the air-particle coupling and track particle deposition, is typically preferred for studying the movement of smoke or BPs. Further, by combining RANS with the Lagrangian particle-tracking model, the rapid prediction of a pollutant’s dispersion and deposition with reasonable accuracy can be obtained, enabling a quicker response to urban biosafety incidents.

Unlike most other pollutants, however, the transmission of toxic BPs is contagious and pathogenic. Normally, BPs are composed of dust, microorganisms, and microbial debris whose particle sizes can range from 0.001 nm to 100 μm. Once inhaled, BPs are eventually deposited in various parts of the human body via the lungs and circulatory system, which not only may cause allergic reactions but also certain diseases^16–20^. In a leakage incident, once high concentrations of BPs spread into an urban environment, it becomes difficult to detect and monitor the situation and risk in a short time because of the odorless and microbial activity characteristics of BPs. Therefore, it is imperative to robustly evaluate the infection risk and evacuate the residents in surrounding urban areas immediately after the leakage of BPs. Based on the calculated wind field and pollutant dispersion, the infection risk can be assessed and appropriate evacuation planning performed. The shortest path algorithms include Dijkstra’s algorithm, the Bellman–Ford algorithm, and the Floyd–Warshall algorithm, all of which have been applied to solve the escape routes of fires, floods, gas leaks, and other disasters^21^. Dijkstra’s algorithm and its improved upon algorithms were widely used in the detection of toxic gas leaks^22–24^. The Gaussian plume model was employed to calculate the toxic dose inhaled by people when ammonia leaked, and Dijkstra’s algorithm was applied to finding the optimal evacuation path^25^. Numerical simulation and Dijkstra’s algorithm can be combined, for example, to calculate the inhalation dose after the transmission of toxic gases for optimizing the crowd evacuation route^21^. It should be noted that the infection risk of contagious BPs on urban scales is rarely reported on when compared to non-biological pollutants such as smoke or chemical gas. Correspondingly, crowd evacuation planning deserves special care and study given that it is nearly impossible to contain the fast transmission of BPs once their leakage occurs.

To sum up, numerical modeling of BPs’ dispersion in the urban environment is crucial but yet not fully investigated so far, whose associated infection risk and evacuation method are of paramount concern for an effective emergency response. This knowledge gap motivated the present numerical investigation of a BP leakage incident from a vaccine factory and the corresponding influence on its surrounding urban environment. The vaccine factory in question is in Baoding City, located on the North China Plain and just over 100 km from Beijing, the capital of China. The model domain has a 520 m × 550 m section, and extends 112 m in the vertical direction with a double-building commercial building. The selected domain consists of buildings varying morphologically in both horizontal and vertical directions. Most of the buildings have a south-north orientation, a typical architectural feature in northern China. This study involves the wind aerodynamics, bioaerosol dispersion and deposition on building surfaces under different thermal conditions, and leakage rates. Combined with the infection risk at pedestrian level assessed by the improved Wells-Riley equation, an approach to conducting the evacuation path planning is proposed based on Dijkstra’s algorithm. This study reveals the transmission mechanism of urban BPs after their accidental leakage and is of guiding significance to biosecurity prevention efforts in urban areas.

## Results

### Airflow patterns under stable and unstable thermal stratification conditions

**Fig. 1:**
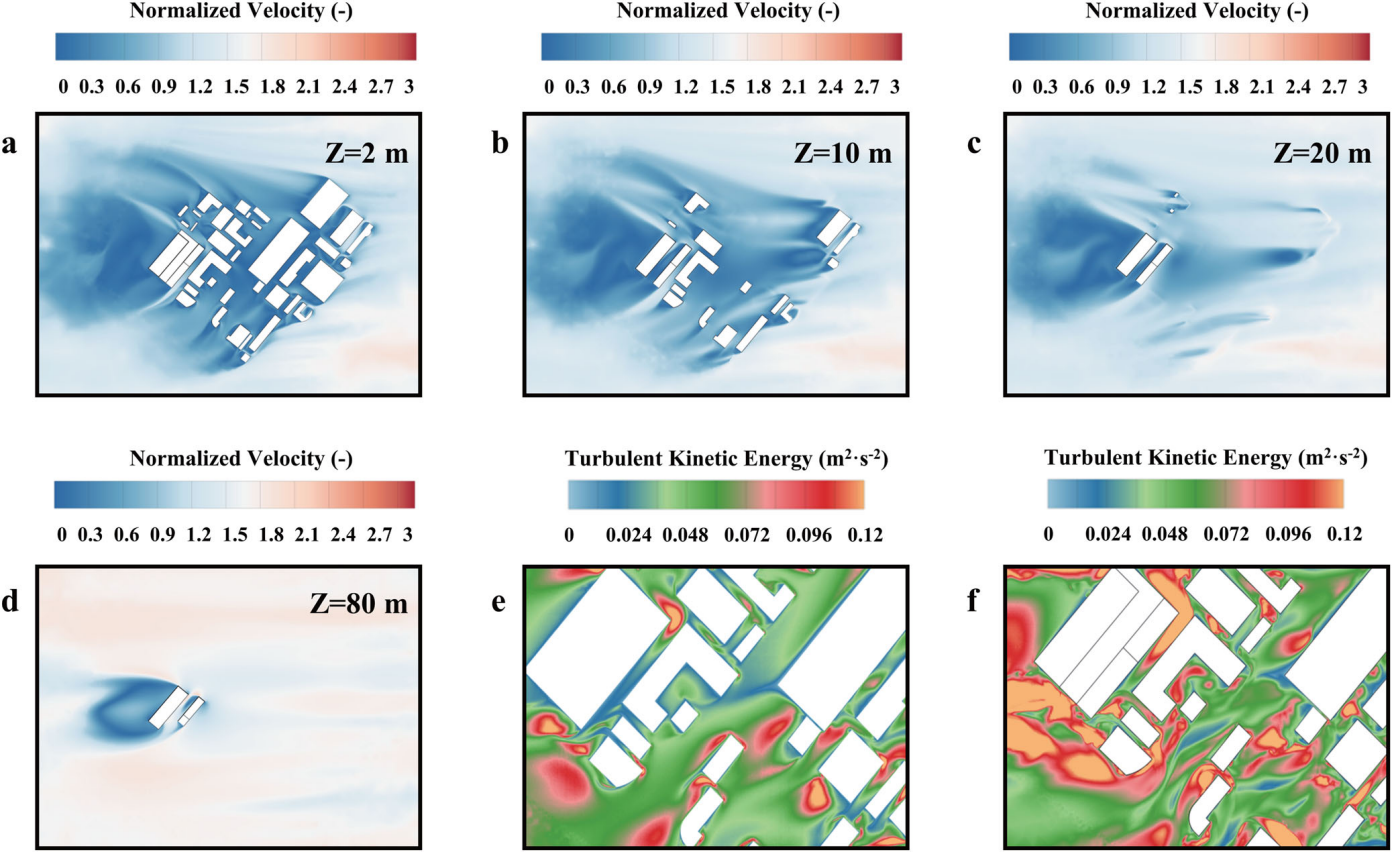
Distribution of airflow pattern around the vaccine factory. **a** The normalized velocity magnitude at *Z* = 2 m for Case Stb-1. **b** The normalized velocity magnitude at *Z* = 10 m for Case Stb-1. **c** The normalized velocity magnitude at *Z* = 20 m for Case Stb-1. **d** The normalized velocity magnitude at *Z* = 80 m for Case Stb-1. **e** The turbulent kinetic energy at *Z* = 1.6 m for Case Stb-1. **f** The turbulent kinetic energy at *Z* = 1.6 m for Case Uns-1.

Airflow is the main factor affecting the spatial dispersion of BPs, so flow dynamics around the vaccine factory were analyzed. The contour of velocity normalized by *U*_ref_ at different heights of the study area in Case Stb-1 is illustrated in Fig. 1. Due to the blocking effect, upward as well as sideward airflow can be observed when the airflow hits the upstream buildings. Wake flows in the form of vortices are generated when airflow passes through the buildings. Evidently, the shedding vortices representing the turbulent flux generate a significant impact on the downstream high-rise buildings up to 80 m in height. Beyond that height, the impact of the upstream vortices due to the blocking of upward airflow gradually decreases.

To clarify the influence of thermal stability upon airflow conveying capacity, the turbulent kinetic energy at the pedestrian level (i.e., *Z* = 1.6 m) was analyzed under stable thermal stratification or unstable thermal stratification. It can be seen that the values for turbulent kinetic energy indicate the airflow tends to be weaker in Case Stb-1 than Case Uns-1. Moreover, the heating effect of the ground and building surfaces contributed to the augmented turbulent kinetic energy in Case Uns-1. Driven by the buoyancy force, the turbulence and mixing of the vortices are strengthened in several areas, including the narrow streets, building leeward areas, and street corners, under the unstable thermal condition. Meanwhile, the airflow in Case Uns-1 seems to have a stronger conveying capacity.

### Bioaerosol deposition on building surfaces

**Fig. 2:**
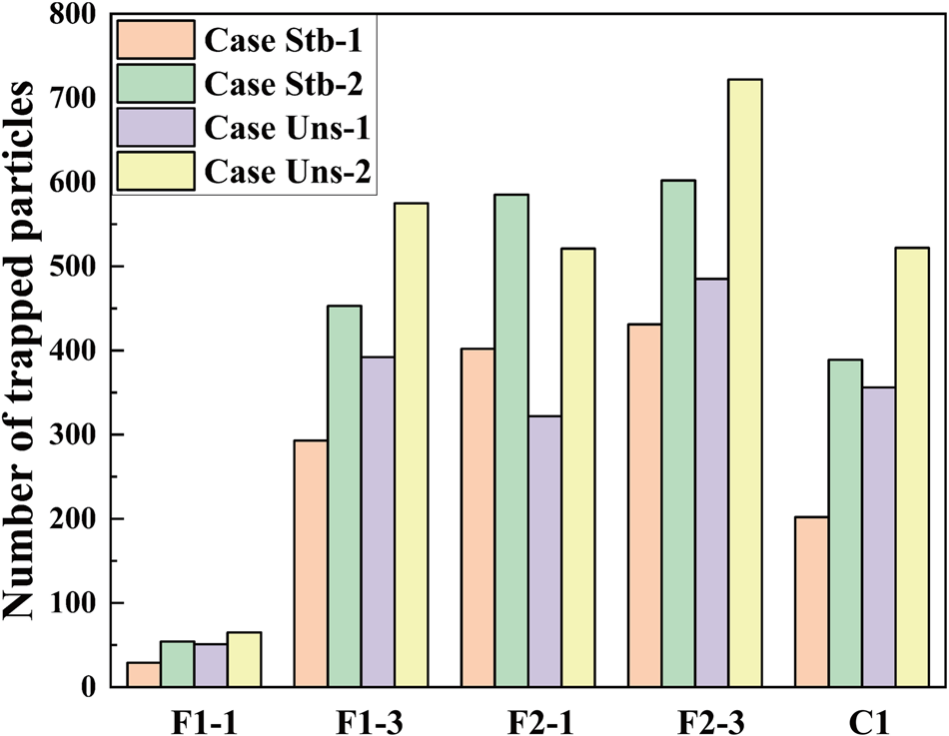
Deposition behaviors of the BPs on typical walls. Number of trapped particles on wall F1-1, F1-3, F2-1, F2-3 and C1 of four cases in the research area.

The BPs deposited on building surfaces are very likely to enter the interior of the building through its windows, causing the infection of indoor personnel. Therefore, the deposition of BPs poses a serious potential threat to both the outdoor and indoor environment. The deposition of BPs on typical walls of the buildings at 900 s since particle release is depicted in Supplementary Fig. 1, while the particle amounts deposited on the walls for four cases are illustrated in Fig. 2. For the high-rise buildings F1 and F2, the BPs are mainly deposited on the right side of the windward wall F2-3, F2-1, and F1-3, where the deposition of BPs can occur at a height of about 80 m (23rd floor) for wall F2-3 and 70 m (19th floor) for wall F1-3. Influenced by the north-to-south building orientation, the airflow first hits the right side of wall F2-3, and then flows around building F2 with relatively uniform deposition of BPs on its left sidewall. Due to the blocking of building F2, the maximum height of BPs’ deposition on wall F2-1 and F1-3 are slightly reduced compared to that on wall F1-3. Those BPs reaching the leeward wall F1-1 are mainly deposited in the upper left corner but in minute quantities. Because the main and secondary vortex generated behind building F1 lie far from wall F1-1, the concentration of BPs is diluted, resulting in a much lower attachment BPs to wall F1-1 vis-à-vis the other walls. This result agrees well with the flow pattern described above, and proves the blocking effect of high-rise building on the upstream vertical distribution of BPs.

Comparing the particle amounts deposited on the walls, the number of particles deposited on the leeward sidewall F2-1 exceeds that on wall F1-3 under the stable thermal condition. For the cases under the unstable thermal condition, only the number of BPs deposited on the leeward side F2-1 is decreased; this is because the thermophoretic force generated by the high ground temperature has a greater influence on BPs’ deposition on the leeward side F2-1. Yet, the largest number of BPs got deposited on wall C1 rather than wall F2-1. This indicated that, compared with Case Stb-1 and Stb-2, more BPs are carried by the airflow to the M + direction in Case Uns-1 and Uns-2 as driven by the thermophoretic force. In addition, the deposition range of BPs on these walls along the M + direction is also enlarged in Case Uns-1 and Uns-2. To sum up, an unstable thermal stratification contributes to increasing the dispersion of BPs in the M + direction in general. Increasing the leakage rate leads to more deposited particles but similar patterns of particle distribution.

### Risk of infection at pedestrian level

**Fig. 3:**
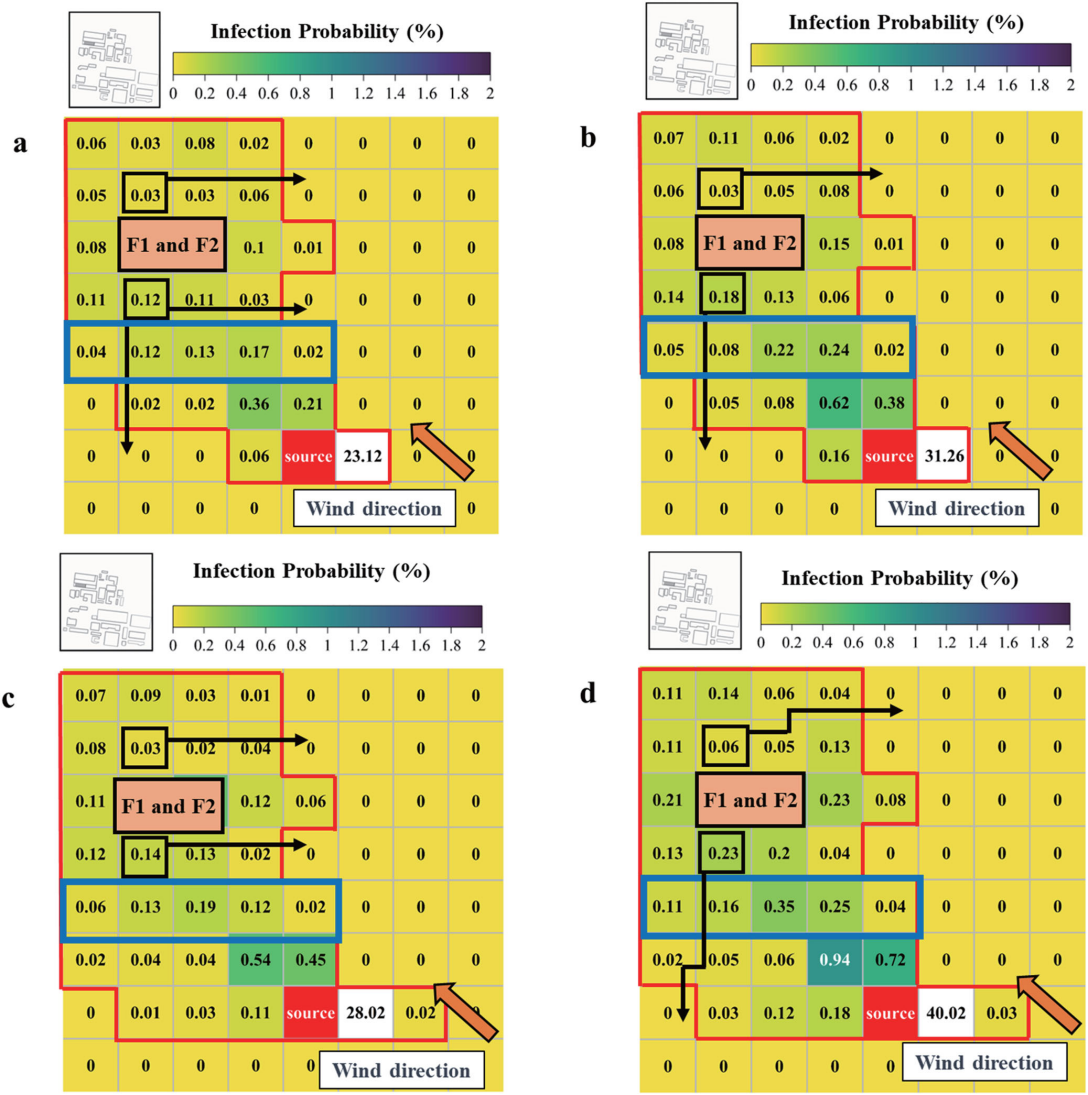
Infection probability and evacuation path at 900 s since particle release at the pedestrian level (*Z* = 1.6 m). **a** Infection probability and evacuation path for Case Stb-1. **b** Infection probability and evacuation path for Case Stb-2. **c** Infection probability and evacuation path for Case Uns-1. **d** Infection probability and evacuation path for Case Uns-2.

To gauge and classify the risk of infection for urban areas, the study area at *Z* = 1.6 m was divided into equal subareas (68.75 m × 65.00 m). For each, its area-averaged concentration of BPs was calculated. Next, the improved Wells-Riley equation was used to evaluate the risk of infection, whose results at 900 s since particle release are presented in Fig. 3. The middle street is marked with a blue box in the figure, and the wind direction is also defined. In all four cases, the highest infection risk can be found in the area next to the pollutant source, which can reach 28.02% for low leakage-rate cases and 40.02% for high leakage-rate cases. Except for the relatively high infection risks in the middle street’s center, the infection risk in each area declines when going from the upstream to downstream along the wind direction. Compared with Case Stb-1 and Stb-2, the cumulative infection risk in the upstream part of the study area for Case Uns-1 and Uns-2 can increase by 5.53% and 9.92%, respectively. In fact, both the infection risk and the infected area under the unstable thermal condition are enlarged, surpassing those under the stable thermal condition, which agrees well with the BPs’ actual dispersion.

Also, the area incurring the highest infection risk in the middle street in Case Uns-1 and Uns-2 moves left in comparison with Case Stb-1 and Stb-2, which is consistent with distribution of increasing concentration of BPs in the M + direction of the middle street. This suggested that the wake flow shedding and mixing driven by the unstable stratification could carry the BPs further to downstream. Besides, the high-rise twin buildings’ blocking effect does lead to a local increase of infection risk in the front of them. However, that higher risk still does not exceed that of the middle street’s areas and poses a relatively low danger to the urban population at the pedestrian level. In sum, those areas distinguished by high infection risk are mainly located 200–300 m upstream from the vaccine factory and the effective infection distance can reach 500 m, similar to the contagion distance of the Brucella leak in Lanzhou (http://wjw.lanzhou.gov.cn/art/2020/9/15/art_4531_928158.html, in Chinese).

### Evacuation path planning

**Fig. 4:**
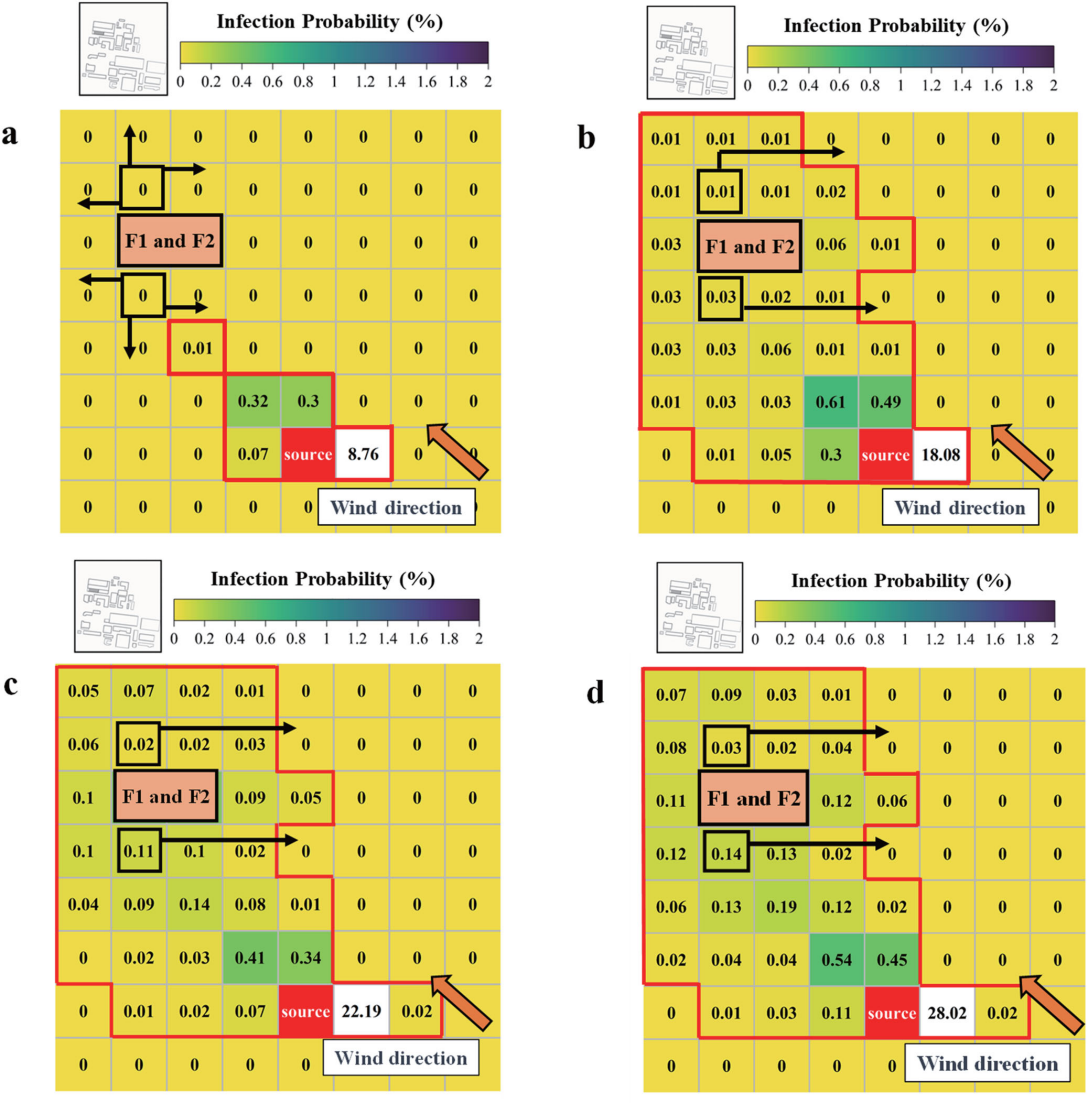
Infection probability and evacuation path at different particle release times at the pedestrian level (*Z* = 1.6 m). **a** Distribution of infection probability and evacuation path at *t* = 5 min. **b** Distribution of infection probability and evacuation path at *t* = 10 min. **c** Distribution of infection probability and evacuation path at *t* = 15 min. **d** Distribution of infection probability and evacuation path at *t* = 20 min.

Enacting a timely emergency response to the leakage of BPs is instrumental in planning the evacuation path of personnel. In this study, combined with the infection risk assessment via the improved Wells-Riley equation, Dijkstra’s algorithm was applied to predict the evacuation paths for several densely populated areas. The calculated evacuation path for the four cases, with their chosen starting points being the school area and high-rise building area, are depicted in Fig. 4. When compared with Case Uns-1 and Uns-2, it is clearly much faster to escape from the high-risk areas in Case Stb-1 or Stb-2 due to the latter’s smaller range of BPs’ dispersion. Naturally, it is preferable that an escapee runs toward the upstream rather the downstream regions. Considering the high infection risk in the middle street, it is prudent to avoid the middle street area along the M + direction and to evacuate towards the wind vent as soon as possible.

To clarify the relationship between exposure time and evacuation path, the infection probability and evacuation path at the pedestrian level at different times were investigated. Case Uns-1 is taken as the representative scenario due to its larger impact range and higher infection risk. As Fig. 4a shows, at *t* = 5 min post-release of BPs, only a few places close to the vaccine factory are exposed to the leaked BPs, leaving the people in the densely populated areas with many evacuation options. But as the exposure time becomes longer, the cumulative infection probability for regions along the transmission path of BPs gradually increases, as seen in Fig. 4b–d. Their corresponding evacuation routes, however, are generally similar since the concentration of BPs in each region tends to be stable. This demonstrates that combining the improved Wells-Riley equation and Dijkstra’s algorithm can provide an effective tool for the evacuation of personnel when the large-scale leakage of BPs occurs in the future.

## Discussion

A simplified modeling of a vaccine factory and its surrounding areas was performed based on field-measured data and Google Maps. Then, the dispersion of BPs under various thermal conditions and leakage rates were investigated. The improved Wells-Riley equation was used to evaluate the infection risk of contagious bioaerosols and Dijkstra’s algorithm applied to guide the optimal escape route.

An unstable thermal stratification contributes to greater turbulence and mixing around the buildings compared with that arising under stable thermal stratification, resulting in a larger range of transmissible high-concentration bioaerosols along the wind direction at the pedestrian level. However, the street canyon under the unstable thermal condition harbors a greater conveyance capacity of bioaerosols. Meanwhile, the blocking effect of the high-rise buildings among the building complex is proven to dominate the vertical distribution of bioaerosols. The surfaces between tall buildings in close proximity may have more bioaerosols deposited on them. Bioaerosol dynamics perpendicular to the mainstream direction, driven by the geometry-induced vortices, contributes more to bioaerosol deposition on sidewalls under an unstable thermal condition. It is recommended that bioaerosol transmission from the outdoor to indoor environment should be paid special attention to for building complexes with a similar layout. Concerning the infection risk, the areas of high infection risk are mainly located 200–300 m upstream but the effective infection distance can reach as far as 500 m from the vaccine factory. Increasing the leakage rate leads to a higher risk of infection but a similar distribution of high-risk regions. When planning the evacuation path, it is advised to avoid the middle street and evacuate towards the wind vent as soon as possible.

The present work may contribute to guiding the urban planning and design of industrial facilities with the potential for pollutant leakage near densely populated areas. It should be noted that the evacuation method examined in this study focused primarily on determining the evacuation route for a specific location at an arbitrary time, assuming a constant walking speed. In the future, the capability of that evacuation method will be explored further, to consider more factors that could affect it, such as pre-evacuation time considerations and variation in human behavior.

## Methods

### Site configuration and boundary conditions

For the research subject, the vaccine factory is marked with a black frame and the school is marked with a red current in Supplementary Fig. 2. The two commercial-type buildings are labeled Building F1 and F2, respectively; building F2 is a polygonal high-rise building, and building F1 is a cube building behind F2. The southeast wind is the dominant wind direction in the vaccine factory region under the typical days of interest, according to meteorological data provided by the Baoding Meteorological Bureau. Meanwhile, the densely populated areas, including shopping malls, commercial buildings, a school, and other places, are localized northwest of the vaccine factory. Once a bioaerosol leakage occurs, it could lead to disastrous consequences for public health; hence, investigating the leakage of BPs under the southeast wind conditions could guide the future design of urban construction and evacuation planning. Thus, the southeast wind constituted the main influencing factor in this study. A release source for BPs was set at the production workshop of the vaccine factory, marked in the red in Supplementary Fig. 2. Due to the transmission effect of wind, a surface source of BPs’ leakage may lead to both advection and diffusion, which could pose a serious threat to people in the whole urban area. To evaluate the deposition of BPs on the building surfaces, typical walls of the buildings are depicted in Supplementary Fig. 2. Wall F2-3 and F2-1 are the windward and leeward sides of building F2. Likewise, walls F1-3 and F1-1 are the windward and leeward sides of building F1. The rest of the smaller typical walls are located in a low-rise building in front of the commercial building. Positioned from left to right are C1, B3, B1, A3, A2, and D1 for low-rise buildings, with the middle street indicated by the blue line. The area below the blue line is defined as the upstream wind direction; conversely, the area above the blue line is defined as the downstream wind direction. In the middle street, the M– and M + directions of the blue line correspond to downstream and upstream of the wind direction, respectively.

Based on Google Maps and derived site measurements, Sketchup Pro software was used to model the study area in its full-scale size. As depicted in Supplementary Fig. 3, the size of densely built-up area is about 520 × 550 × 120 m. The negative direction of the *X*-axis is the airflow direction, the *Z*-axis is the vertical direction, and the *Y*-axis is the horizontal direction. Following the recommendation in the CFD guideline^26^ for urban area modeling, the distance from the building complex to the domain outlet is 20 *H*, where *H* is defined as the height of the tallest building. In the other directions, the distance between the building complex and the boundaries are all set to 5 *H*. The details on the spatial discretization were depicted in Supplementary Methods and Supplementary Fig. 4.

The boundary condition of the velocity inlet was applied at the domain inlet. Symmetry boundary conditions were imposed for the sides and top of the model, with outflow used for the outlet. The Boussinesq approximation was employed to address the temperature-induced density variation in air. The operating density of air is 1.225 kg·m^−3^. The specific heat *C*_p_ is 1006.43 J·kg^−1^·K^−1^ and the thermal conductivity is 0.0242 W·m^−1^·K^−1^. The thermal expansion coefficient of air is 0.00347 K^−1^. Wind speed at the inlet *U*(*Z*) was defined as follows:

1 $$U\left( Z \right) = U_{{{{\mathrm{ref}}}}}(Z/H_{{{{\mathrm{ref}}}}})^\alpha$$

where *U*_ref_ = 1.773 m·s^−1^ is the annual mean wind speed at the height *H*_ref_ = 10 m, this based on the relevant meteorological data provided by the Baoding Meteorological Bureau. According to urban characteristics and the building density, the value of *α* was set to 0.3^27^. To fully consider the environmental conditions of the studied urban area of interest here, two scenarios differing in thermal conditions, as represented by different average air and ground temperatures, were examined. For this, the bulk Richardson number, describing the ratio of buoyancy to inertial forces, is defined as:

2 $${{{\mathrm{Rb}}}} = gH_{{{{\mathrm{ref}}}}}\left( {T_{{{{\mathrm{in}}}}} - T_{{{\mathrm{g}}}}} \right)/\left( {T_{{{\mathrm{a}}}} + 273} \right)\left( {U_{{{{\mathrm{ref}}}}}} \right)^2$$

where *g* denotes the gravitational acceleration; *T*_a_ denotes the ambient air temperature of the freestream; and *U*_ref_ denotes the horizontal velocity at *Z* = *H*_ref_, which varies for different Rb conditions. *T*_in_ is the inlet air temperature and *T*_*g*_ is the wall temperature. In Case Stb-1, the air temperature was set to 28.1 °C and the wall temperature set to 15 °C, resulting in a stable thermal stratification with a bulk Richardson number of 1.35. In Case Uns-1, the air temperature was set lower, to 17 °C, and the wall temperature set higher, to 20 °C, resulting in an unstable thermal stratification with a bulk Richardson number of –0.57.

### CFD setup and dispersion model

For the numerical simulation, the commercial CFD software Fluent 2021R1 was employed. Although high-fidelity turbulence models including LES or DES models are preferable when seeking high calculation accuracy, huge simulation resources and computing time are required. Actually, good agreement with the experimental results can be obtained by both the RANS and LES models for pollutant dispersion^28^. It is widely recognized that RANS can serve as a robust research tool in the study of urban environments and pollutants’ dispersion^29–32^. As such, the Realizable *k-ε* model was used in the present work for resolving turbulence^33^. The enhanced wall function was selected near the wall of each building given its ability to resolve the thermal effect near walls^34^. The governing equations were discretized using the finite volume method and applying a second-order upwind style. The general form of the Realizable *k-ε* governing equation is as follows:

3 $$\partial \left( {\rho \varphi } \right)/\partial t + \nabla \cdot \left( {\rho \varphi {{{\vec{\mathbf V}}}}} \right) = \nabla \cdot \left( {{\Gamma}_\varphi \nabla \varphi } \right) + {{{\mathbf{O}}}}_\varphi$$

where *ρ* is the air density, $\overrightarrow {{{\mathbf{V}}}}$ denotes the air velocity vector, *φ* represents each of the three velocity components, ${\Gamma}_\varphi$ is the effective diffusion coefficient of *φ*, and ${{{\mathbf{O}}}}_\varphi$ is the source term. The pressure–velocity coupling was solved by the Semi-Implicit Method for Pressure Linked Equations Consistent (SIMPLEC), based on an iterative process that included solving the pressure correction equation to ensure mass conservation. The least-squares method of gradient discretization was implemented, and the PRESTO format used for pressure discretization. Furthermore, the momentum, energy, *k*, and *ε* equations were also discretized by using the second-order upwind.

The discrete phase model adopts the Lagrangian discrete random walk model to track the dispersion path of BPs in urban areas. The governing equation is defined as:

4 $$d\vec u_{{{\mathrm{p}}}}/dt = F_{{{\mathrm{D}}}}\left( {\overrightarrow {\it{u}} - \overrightarrow {{\it{u}}_{{{\mathrm{p}}}}} } \right) + \overrightarrow g (\rho _{{{\mathrm{p}}}} - \rho )/\rho _{{{\mathrm{p}}}} + \overrightarrow F$$

where $\overrightarrow u$ and $\overrightarrow {u_{{{\mathrm{p}}}}}$ denote the velocity of airflow and BPs, respectively; *F*_*D*_ represents the drag forces acting on the BPs; *ρ* and *ρ*_*p*_ is the density of airflow and BPs, respectively; $\overrightarrow g$ is gravitational acceleration; and $\overrightarrow F$ represents additional forces acting upon BPs. The validations of the simulation models were performed with wind tunnel data (see Supplementary Fig. 5 and Supplementary Methods).

A computing workstation housing a 48-core CPU of AMD EPYC 7R32 was used for simulation, whose total calculation time was about 240 h. For this simulation, the steady-state flow field was calculated. A scaled residual value of 10^−8^ was used to measure the calculation convergence. The imbalance error of the total mass and energy between the domain inlet and outlet in the simulation was guaranteed to be below 1%. Next, the unsteady simulation was with the particles injected. The time step of the unsteady calculation was set to 1 s, and each time step was iteratively calculated 20 times. In order to consider the influence of thermal conditions on BPs, the Saffman lift force and thermophoresis force were incorporated in the discrete phase setup. The release source of BPs was selected, at the window of the production workshop, this having a size of 1.5 m × 1.2 m, with a normal direction of surface release selected to inject particles. The BPs were released at a speed of 1 m·s^−1^, for which the release temperature was assumed to be the same as air temperature. The particle size of the BPs was set to 0.5 μm. According to their emission concentration after undergoing disinfection by the vaccine factory, the source concentration of BPs was set to 3.6 × 10^−7^ kg·s^−1^ in Case Stb-1 and Case Uns-1. To clarify the effects of leakage rate on the emergency evacuation, the source concentration was set to 7.2 × 10^−7^ kg·s^−1^ in both Case Stb-2 and Case Uns-2. The simulation time was set to 20 min because the monitored concentration changed little when the simulation time was longer than 820 s. Both the wall and the ground were designated as trap boundary conditions, and the possibility that any BPs falling on the wall may enter the building through the window was also considered in each simulation.

### Infection risk model and evacuation path planning

The Wells-Riley equation has a wide range of applications in assessing the risk of BPs^35^. For example, it has been used to assess the risk of viral infection between different high-rise residential buildings^15^. In the present study, an improved Wells-Riley equation was applied to assess the risk of infection at outdoor pedestrian level height of *Z* = 1.6 m in urban areas^36^. The governing equation is as follows:

5 $$P = 1 - e^{ - pCt}$$

where *P* is the probability of spatial infection event occurring; *p* is the adult respiratory rate, this taken to be 1.222 m^3^·h^−1^^37^ *C* is the bioaerosol concentration (kg·m^−3^); and *t* is the bioaerosol residence time (h). Because the virus in the vaccine factory was presumed to be highly contagious, the leaked bioaerosol quanta in the simulation was set to 10,000 quanta·h^−1^^15^. The concentration of BPs was normalized with *C*_*S*_ of 1.0 × 10^−11^ kg·m^−3^.

Dijkstra’s algorithm was used to predict the evacuation paths for several densely populated areas. The main feature of this algorithm is that it starts from the a few selected points and adopts a greedy algorithm strategy. Then, the adjacent nodes of the vertices that are closest to the starting point are visited each time until it extends to the endpoint. The pseudocode corresponding to this algorithm is presented in Supplementary Table 1.

## Supplementary information

Supplementary Information

## Data Availability

Other data that support the findings of this study are available from the corresponding author upon reasonable request.

## Code availability

Codes used to generate the results of this study are available from the authors on reasonable request.

## References

[1] Nieuwenhuijsen MJ (2021). New urban models for more sustainable, liveable and healthier cities post covid19; reducing air pollution, noise and heat island effects and increasing green space and physical activity. Environ. Int..

[2] Magnuson M (2014). Analysis of environmental contamination resulting from catastrophic incidents: Part 2. Building laboratory capability by selecting and developing analytical methodologies. Environ. Int..

[3] Mu D, Gao N, Zhu T (2016). Wind tunnel tests of inter-flat pollutant transmission characteristics in a rectangular multi-storey residential building, part A: Effect of wind direction. Build. Environ..

[4] Tan Z, Tan M, Sui X, Jiang C, Song H (2019). Impact of source shape on pollutant dispersion in a street canyon in different thermal stabilities. Atmos. Pollut. Res..

[5] Derudi M, Bovolenta D, Busini V, Rota R (2014). Heavy gas dispersion in presence of large obstacles: Selection of modeling tools. Ind. Eng. Chem. Res..

[6] Tan W, Li C, Wang K, Zhu G, Liu L (2019). Geometric effect of buildings on the dispersion of carbon dioxide cloud in idealized urban street canyons. Process Saf. Environ. Prot..

[7] Zhao Y (2022). Boundary layer wind tunnel tests of outdoor airflow field around urban buildings: A review of methods and status. Renew. Sustain. Energy Rev..

[8] Lauriks T (2021). Application of Improved CFD Modeling for Prediction and Mitigation of Traffic-Related Air Pollution Hotspots in a Realistic Urban Street. Atmos. Environ..

[9] van Hooff T, Blocken B (2010). Coupled urban wind flow and indoor natural ventilation modelling on a high-resolution grid: A case study for the Amsterdam ArenA stadium. Environ. Model. Softw..

[10] Tominaga Y, Stathopoulos T (2013). CFD simulation of near-field pollutant dispersion in the urban environment: A review of current modeling techniques. Atmos. Environ..

[11] Toparlar Y, Blocken B, Maiheu B, van Heijst GJF (2017). A review on the CFD analysis of urban microclimate. Renew. Sustain. Energy Rev..

[12] Ai ZT, Mak CM (2017). CFD simulation of flow in a long street canyon under a perpendicular wind direction: Evaluation of three computational settings. Build. Environ..

[13] Hang J (2017). The influence of street layouts and viaduct settings on daily carbon monoxide exposure and intake fraction in idealized urban canyons. Environ. Pollut..

[14] Zhang Y (2020). Numerical studies of passive and reactive pollutant dispersion in high-density urban models with various building densities and height variations. Build. Environ..

[15] Wu Y, Niu J (2017). Numerical study of inter-building dispersion in residential environments: Prediction methods evaluation and infectious risk assessment. Build. Environ..

[16] Humbal C, Gautam S, Trivedi U (2018). A review on recent progress in observations, and health effects of bioaerosols. Environ. Int..

[17] Ferguson RMW (2021). Size fractionation of bioaerosol emissions from green-waste composting. Environ. Int..

[18] Ching J, Adachi K, Zaizen Y, Igarashi Y, Kajino M (2019). Aerosol mixing state revealed by transmission electron microscopy pertaining to cloud formation and human airway deposition. npj Clim. Atmos. Sci..

[19] Violaki K (2021). Bioaerosols and dust are the dominant sources of organic P in atmospheric particles. npj Clim. Atmos. Sci..

[20] Li Q, Zhang H, Cai X, Song Y, Zhu T (2021). The impacts of the atmospheric boundary layer on regional haze in North China. npj Clim. Atmos. Sci..

[21] Wang J, Yu X, Zong R, Lu S (2022). Evacuation route optimization under real-time toxic gas dispersion through CFD simulation and Dijkstra algorithm. J. Loss Prev. Process Ind..

[22] Fabiano B, Currò F, Reverberi AP, Pastorino R (2005). Dangerous good transportation by road: From risk analysis to emergency planning. J. Loss Prev. Process Ind..

[23] Dou Z (2019). Review on the emergency evacuation in chemicals-concentrated areas. J. Loss Prev. Process Ind..

[24] Gai W, Deng Y, Jiang Z, Li J, Du Y (2017). Multi-objective evacuation routing optimization for toxic cloud releases. Reliab. Eng. Syst. Saf..

[25] Xu K, Gai WM, Salhi S (2021). Dynamic emergency route planning for major chemical accidents: Models and application. Saf. Sci..

[26] Tominaga Y (2008). AIJ guidelines for practical applications of CFD to pedestrian wind environment around buildings. J. Wind Eng. Ind. Aerodyn..

[27] Liquide A, Systems M (2016). Tech. note Tech. note.

[28] Qu Y, Milliez M, Musson-Genon L, Carissimo B (2012). Numerical study of the thermal effects of buildings on low-speed airflow taking into account 3D atmospheric radiation in urban canopy. J. Wind Eng. Ind. Aerodyn..

[29] Habilomatis G, Chaloulakou A (2015). A CFD modeling study in an urban street canyon for ultrafine particles and population exposure: The intake fraction approach. Sci. Total Environ..

[30] Tominaga Y, Stathopoulos T (2018). CFD simulations of near-field pollutant dispersion with different plume buoyancies. Build. Environ..

[31] Hao C, Xie X, Huang Y, Huang Z (2019). Study on influence of viaduct and noise barriers on the particulate matter dispersion in street canyons by CFD modeling. Atmos. Pollut. Res..

[32] Tominaga Y, Stathopoulos T (2017). Steady and unsteady RANS simulations of pollutant dispersion around isolated cubical buildings: Effect of large-scale fluctuations on the concentration field. J. Wind Eng. Ind. Aerodyn..

[33] Tauseef M, Rashtchian D, Abbasi A (2011). CFD-based simulation of dense gas dispersion in presence of obstacles. J. Loss Prev. Proc..

[34] Mu D, Gao N, Zhu T (2018). CFD investigation on the effects of wind and thermal wall-flow on pollutant transmission in a high-rise building. Build. Environ..

[35] Liu M (2022). Evaluation of different air distribution systems in a commercial airliner cabin in terms of comfort and COVID-19 infection risk. Build. Environ..

[36] Ruggiero L, Ronchi E, Maragkos G, Beji T, Merci B (2016). A dynamic approach for the impact of a toxic gas dispersion hazard considering human behaviour and dispersion modelling. J. Hazard Mater..

[37] Liu Z (2021). Potential infection risk assessment of improper bioaerosol experiment operation in one BSL-3 laboratory based on the improved Wells-Riley method. Build. Environ..

