# Supplementary material for: Modeling the infection risk and emergency evacuation from bioaerosol leakage around an urban vaccine factory
Source: NPJ Clim Atmos Sci. 2023 Feb 17;6(1):6. doi: 10.1038/s41612-023-00342-1 (PMC9937520; doi:10.1038/s41612-023-00342-1)
Supplement: Supplementary file 1 — Supplementary Information [file 41612_2023_342_MOESM1_ESM.pdf]

Supplementary Information for "Modeling the infection risk and emergency evacuation from bioaerosol leakage around an urban vaccine factory"

Zhijian Liu[a,*], Hongwei Cao[a], Chenxing Hu[b,*], Minnan Wu[a], Siqi Zhang[a], Junzhou He[a], Chuan Jiang[a]

[a]School of Energy and Power Engineering, North China Electric Power University, Baoding 071003, China

[b]School of Mechanical Engineering, Beijing Institute of Technology, Beijing 100081, China

*Corresponding authors: zhijianliu@ncepu.edu.cn; 6120210154@bit.edu.cn

SUPPLEMENTARY FIGURES

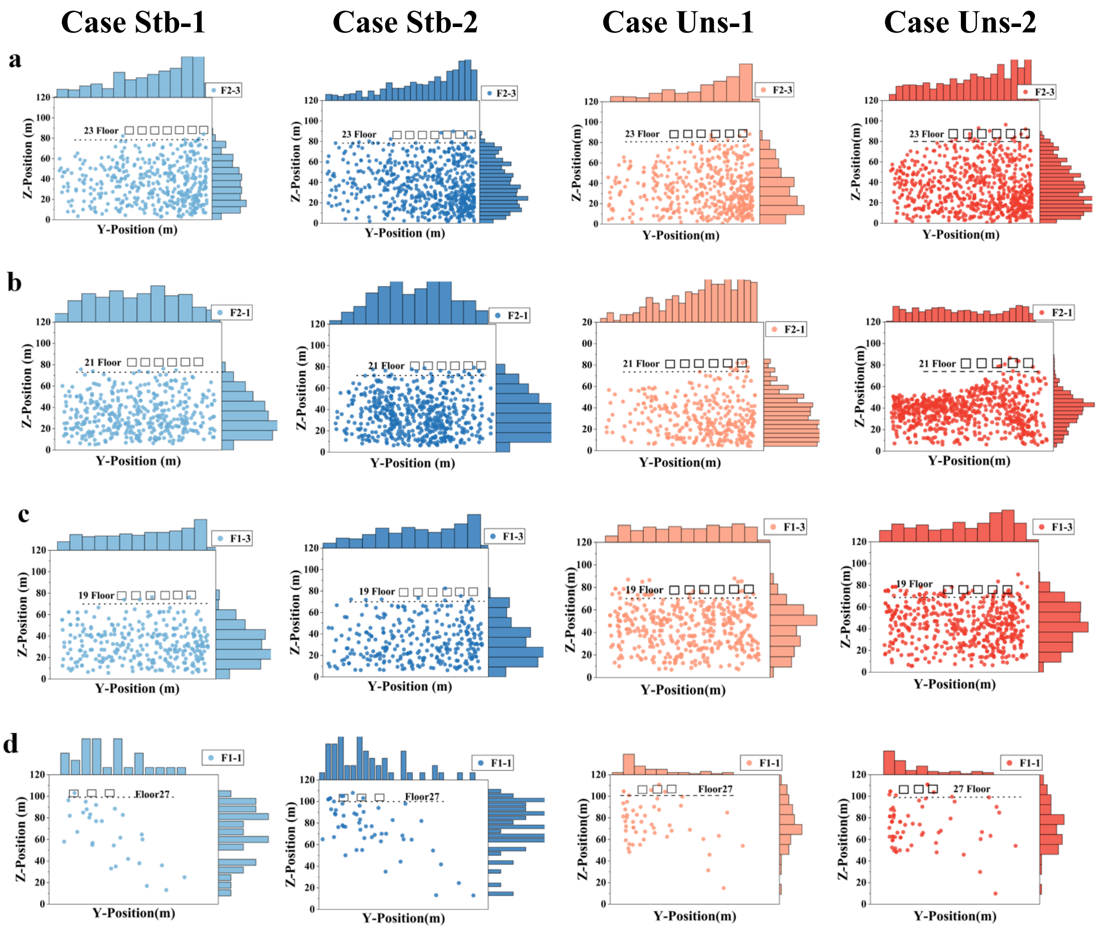

Supplementary Figure 1 Bioaerosol particle deposition locations on typical walls of four cases. a Distribution of deposited BPs on F2-3 wall. **b** Distribution of deposited BPs on F2-1 wall. **c** Distribution of deposited BPs on F1-3 wall. **d** Distribution of deposited BPs on F1-1 wall.

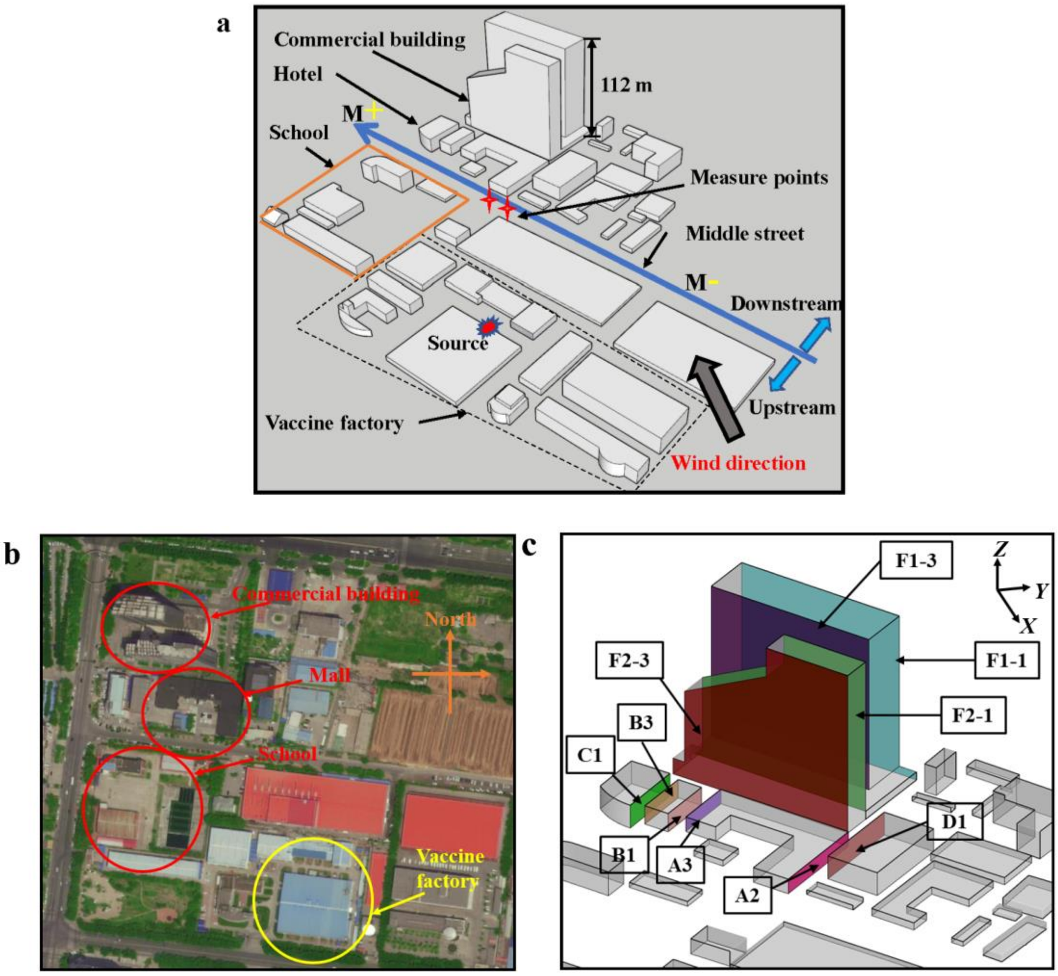

Supplementary Figure 2 Schematic diagram of the research area. a Layout of buildings in the study area. **b** Satellite map of the study area. **c** Location of typical walls.

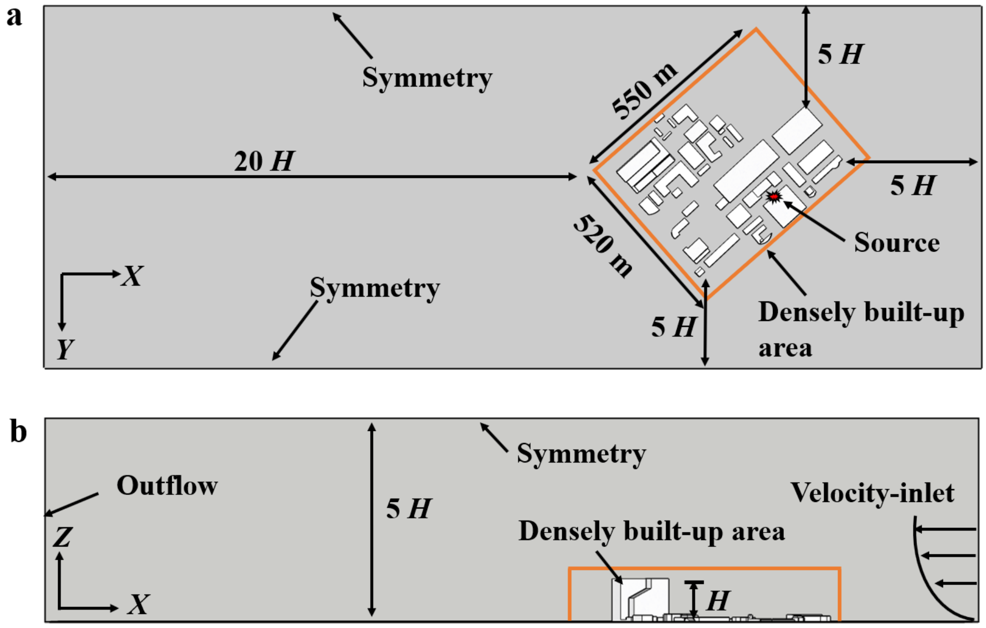

Supplementary Figure 3 Schematic diagram of the computational domain. a Top view and

boundary conditions of the computational domain. **b** Side view and boundary conditions of the computational domain.

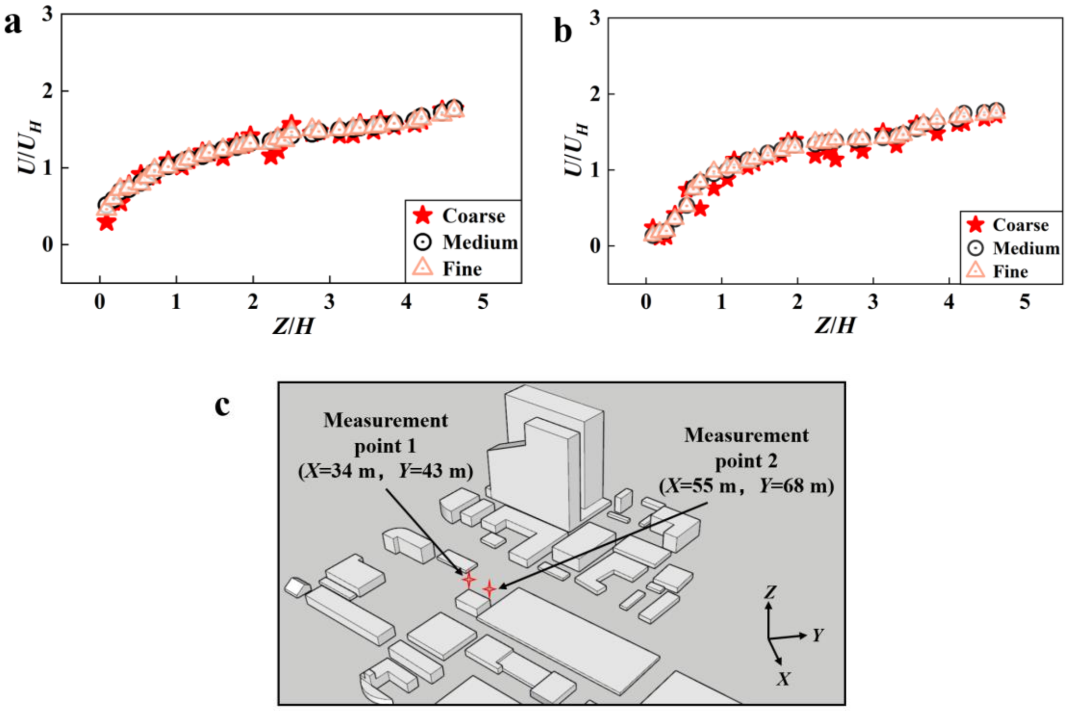

Supplementary Figure 4 Grid number independence verification with three mesh resolutions. **a** Distribution of normalized velocity along vertical direction at measurement point 1. **b** Distribution of normalized velocity along vertical direction at measurement point 2. **c** Schematic diagram of the measurement points.

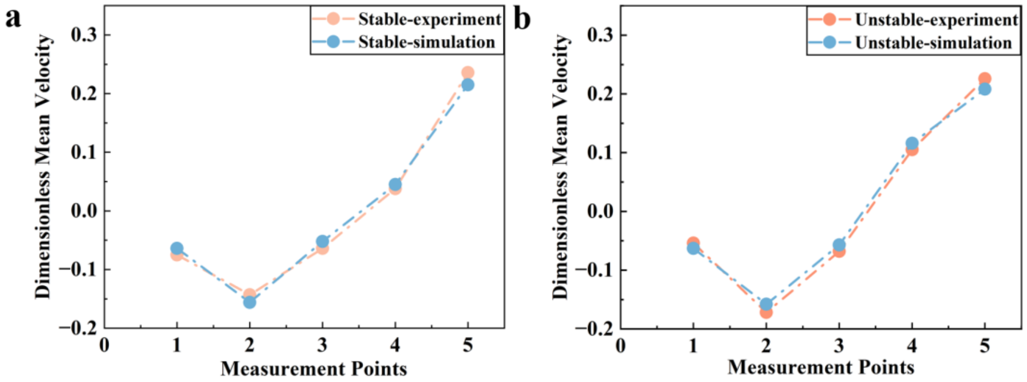

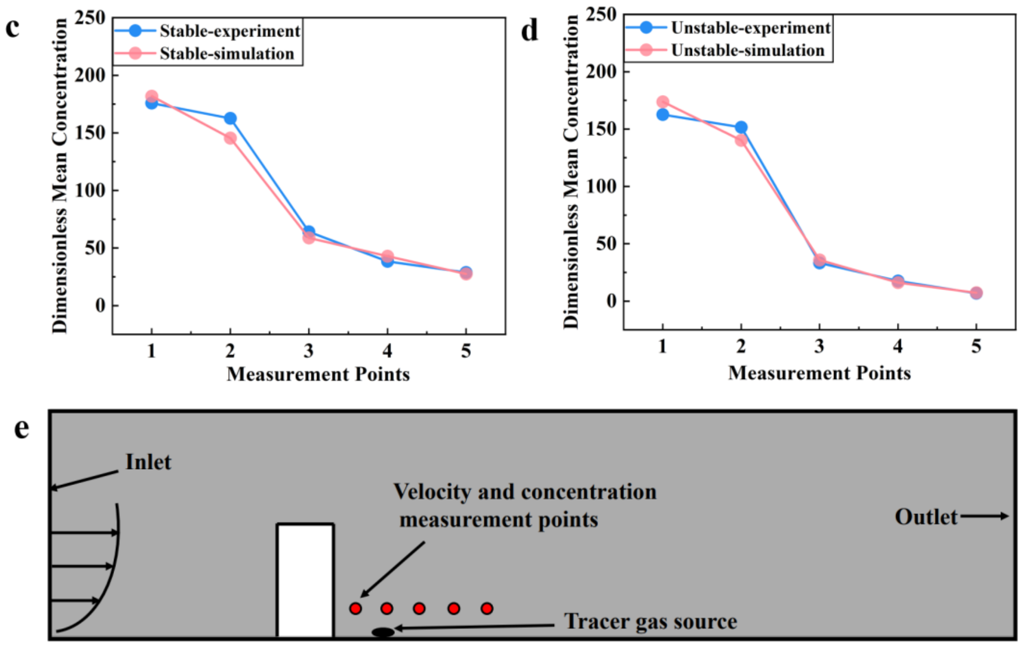

Supplementary Figure 5 Validation of simulated velocity and concentration against wind tunnel data. a Dimensionless mean wind velocity under a stable thermal condition. **b** Dimensionless mean wind velocity under an unstable thermal condition. **c** Dimensionless mean concentration under a stable thermal condition. **d** Dimensionless mean concentration under an unstable thermal condition. **e** Sketch of the measurement points at wind tunnel.

SUPPLEMENTARY TABLES

Supplementary Table 1 Pseudocode corresponding to Dijkstra's algorithm.

Algorithm
$S \leftarrow$ *set of sources*<br>$s \leftarrow$ *element in source set*<br>$V \leftarrow$ *set of vertices*<br>$v \leftarrow$ *element in vertice set*<br>$Prob \leftarrow$ *cumulative infection probability*<br>$w \leftarrow$ *infection probability of each vertice*<br>$S \leftarrow \{s\}$
$Prob[s,s] \leftarrow 0$
**for** $v_i \in V - \{s\}$ **do**
$Prob[s,v_i] \leftarrow w(s,v_i)$ $(when\ v_i\ not\ found, Prob[s,v_i] \leftarrow \infty)$
**while** $V - S \neq \emptyset$ **do**
$find\ min\ Prob[s,v_i]\ from\ the\ set\ V - S$
$S \leftarrow S \cup \{v_j\}$
**for** $v_i \in V - S$ **do**
**if** $Prob[s,v_j] + w_{j,i} < Prob[s,v_i]$ **then**
$Prob[s,v_i] \leftarrow Prob[s,v_i] + w_{j,i}$

SUPPLEMENTARY METHODS

Grid independence verification

The commercial meshing software of ICEM CFD was used to build the unstructured mesh in this study. For the densely built-up area, the maximum grid size was 0.8 m. Further, given the importance of considering the thermal effect, the grid was refined in the near-wall regions of interest (namely, close to the building groups) and the ground boundary. To guarantee the use of an enhanced wall treatment, The mesh size of the first layer was 0.002 m and the dimensionless wall distance y^+ for near-walls was be below 5. To verify the grid independence of the simulation, three mesh resolutions—coarse mesh (total grid number: 5.31 million), medium mesh (total grid number: 36.80 million) and fine mesh (total grid number of 53.63 million)—were tested at two positions. The test points were localized on the middle street with the spatial coordinate (X, Y) of (34m, 43m) and (55m, 68m). In the vertical direction, the velocity was measured every 0.15H. The height of the tallest building H was used to nondimensionalize the spatial coordinate in vertical direction. And the velocity at the height of H was used to nondimensionalize the measured velocity. The simulated velocity values of these two points at different heights appear in Supplementary Figure 4. Finally, after considering the calculation efficiency and accuracy of the mesh types, the medium-sized mesh with the total grid number of 36.80 million was selected for the simulation.

Wind tunnel data validations

To validate the simulation methods, wind tunnel experiments around an isolated building were carried out under both stable and unstable thermal stratification conditions by Tokyo University of Technology (http://www.wind.arch.t-kougei.ac.jp/info_center/pollution/Non-Isothermal_Flow.html). As seen in Supplementary Figure 5, the predicted wind velocity at selected measurement points were compared with the wind tunnel data under both stable and unstable thermal stratification conditions. The measurement points localized in the wake zone of the isolated building at pedestrian level were used. The relative error between the simulated

and experimental results under either scenario was below 10%. Hence, this proved the present simulation method is able to ensure the prediction accuracy of the wind fields and thermal effect in the outdoor environment. Furthermore, to validate the method for predicting gas dispersion, the wind tunnel data from the same experiment was employed in gas concentration modeling. Mean concentrations of the released tracer gas were well predicted, in that the relative error was below 5% under both the stable and unstable thermal stratification condition. In summary, the numerical methods used in this study were validated and capable of predicting the wind field, thermal effect, and gas concentration with reasonable accuracy.
